# The Medical Council and Dr. Irvine

**Published:** 1900-12-08

**Authors:** 


The Hospital

A Journal of

The Medical Sciences and Hospital Administration.

Vol. XXIX.—No. 741.] Edited by Sir Henry Burdett, K.C.B., and Solomon C. Smith, M.D., M.R.C.P. [December 8, 1900.

### The Medical Council and Dr. Irvine.

The General Medical Council was occupied, on Thursday of last week in considering a case of considerable importance to the profession, and with regard to which its decision, or rather its avoidance of a decision, has left much to be desired. Dr. Henry Ward Irvine, of Newhall Street, Birmingham, registered as M.B., Bac. Surg. 1895, M.D. 1897, Univ. Dubl., was summoned to answer the following charge, made on the complaint of Dr. Saundby, and formulated by the Council's solicitor:—

That he accepted the office of Consulting Physician to the Consultative Medical and Surgical Institution, Birmingham, at a salary, and approved or acquiesced in the extensive advertisements issued by that institution by posters exhibited in the various manufactories, trading establishments, and mercantile offices of the city, by advertisements in the daily press, and by lithographed letters and printed pamphlets, widely circulated, setting forth conspicuously his name and medical qualifications, and referring to his special ability as a General Consulting Practitioner, and soliciting and inviting the public to consult him at a reduced fee.

The complaint was supported, on behalf of Dr. Saundby, by the Medical Defence Union; and Dr. Irvine was defended by Mr. G. M. Freeman, Q.C., who called as witnesses Mr. Alderman Cook, Chairman of the Hospital Saturday Fund of Birmingham, Mr. Aston, the Honorary Secretary of the "Institution" by which Dr. Irvine was employed, and Mr. Arthur Chamberlain.

The case arose, we need hardly say, out of the proceedings of an institution upon which we have commented on former occasions, and which was said by Mr. Freeman to have been established in order "to provide consultation for the working men at a reasonable fee." Mr. Arthur Chamberlain described it as "a philanthropic concern, which they hoped one day to see self-supporting," and said that he had himself given £200 towards its expenses; while it appeared that the advertisements were issued by the Institution, as a means of obtaining the publicity and support without which the desired independence of outside pecuniary assistance could not be secured. There was no dispute as to any of the essential facts of the case; and the question before the Council was, in reality, whether advertising, apart from anything specially objectionable in the character of the advertisements, constituted conduct which could properly be described as "infamous in a professional respect," and which would therefore fall within the penal jurisdiction of the Council. The affirmative of this proposition has not hitherto been maintained by the Courts, and a legal decision of the question is much to be desired. No better opportunity of obtaining one could have been imagined, but the course actually pursued by the Council has suffered it to be thrown away.

Some years ago, it was the custom of the Council, in deciding upon a charge of "infamous conduct," to vote first on the question whether the conduct imputed had been proved. If this were affirmed, the next question was whether it were infamous. A decision in the negative dismissed the complaint; a decision in the affirmative was necessarily followed by removal from the register, on which, of course, a person found to have been guilty of infamous conduct could not be suffered to remain. Mr. Brudenell Carter, when a member of the Council, suggested and carried a somewhat different way of dealing with cases which presented extenuating circumstances. On this, the method now pursued, when the conduct has been proved, the next question is whether the Council shall at once proceed to pass judgment on the case, or shall adjourn it for future consideration. If the latter alternative be adopted, the Council practically says to the accused: "You have done what you were charged with doing, but we will not at present say that we regard your conduct as infamous. Come again in six months, and show us that you have abandoned the course complained of, and it is possible that we may then take a lenient view." The position, in short, is analogous to that of a Court which directs a person to come up for judgment if called upon; and the course is only applicable to cases in which very young men have been led astray, or in which offences have been due rather to want of consideration than to deliberate wrong-doing. Neither of these conditions was present in the case of Dr. Irvine. He has been for three years a Doctor of Medicine of a distinguished University; he was perfectly aware of the issue of the advertisements; and, instead of expressing regret, or offering excuses, he defended his action before the Council, which, nevertheless, deferred judgment until May. If the Council were of opinion that Dr. Irvine's conduct was "infamous," it was a plain duty to say so, and to remove him from the register. If the Council did not think his conduct "infamous," it was an equally plain duty to say so, and to bring the case to a conclusion. The course actually pursued was merely a weak evasion of the difficulty. It was neither to run with the hare nor to hunt with the hounds. The Council probably hoped to frighten Dr. Irvine into abandoning his appointment, and, at the same time, to avoid committing itself to a decision against him, which, if arrived at, would certainly be, and which ought to be, questioned before the High Court. Mr. Justice Henn Collins, in the Allinson case, declared that advertising by a medical practitioner was not sufficient to constitute infamous conduct, unless there were something objectionable in the character of the advertisements themselves, something which Allinson was afterwards held to have supplied by "defaming his brother practitioners." If this view of the law were superseded, and if it were infamous, and involved legal penalties, for a medical practitioner to announce by public advertisement his name and qualifications and his readiness to attend patients, the change would be a very far-reaching one, the effects of which it might not be easy to foresee. It is even possible, in such a condition of affairs, that the Medical Council might be called upon to sit in judgment upon certain medical publications, or even upon some of the "cures" reported in the columns of our contemporaries. If the law does give this power to the Council, the power should be exercised fearlessly and at once. If not, it is beneath the dignity of the Council to set up a mere bugbear which will cease to be alarming as soon as its character is exposed.

